# Removal of micropollutants through a biological wastewater treatment plant in a subtropical climate, Queensland-Australia

**DOI:** 10.1186/s40201-016-0257-8

**Published:** 2016-11-03

**Authors:** Miguel Antonio Reyes Cardenas, Imtiaj Ali, Foon Yin Lai, Les Dawes, Ricarda Thier, Jay Rajapakse

**Affiliations:** 1 MI Murrumbidgee Irrigation, Research Station Road, Hanwood, NSW 2680 Australia; 2 Treatment Program, Logan City Council, Logan City DC, QLD 4114 Australia; 3 National Research Centre for Environmental Toxicilogy (EnTox), The University of Queensland, Brisbane, QLD 4108 Australia; 4 Science and Engineering Faculty, School of Earth, Environment and Biological Sciences, Queensland University of Technology, QLD 4001 Brisbane, Australia; 5 Faculty of Health, Queensland University of Technology, QLD 4001 Brisbane, Australia

**Keywords:** Biological wastewater treatment, Micropollutants removal, Sub-tropical climate

## Abstract

**Background:**

Municipal wastewaters contain a multitude of organic compounds derived from domestic and industrial sources including active components of pharmaceutical and personal care products and compounds used in agriculture, such as pesticides, or food processing such as artificial sweeteners often referred to as micropollutants. Some of these compounds or their degradation products may have detrimental effects on the environment, wildlife and humans. Acesuflame is one of the most popular artificial sweeteners to date used in foodstuffs. The main objectives of this descriptive study were to evaluate the presence of micropollutants in both the influent and effluent of a large-scale conventional biological wastewater treatment plant (WWTP) in South-East Queensland receiving wastewater from households, hospitals and various industries.

**Methods:**

Based on USEPA Method 1694: Filtered samples were spiked with mass-labelled chemical standards and then analysed for the micropollutants using liquid chromatography coupled with tandem mass spectrometry.

**Results:**

The presence of thirty-eight compounds were detected in the wastewater influent to the treatment plant while nine of the compounds in the categories of analgesic, anti-inflammatory, alkaloid and lipid/cholesterol lowering drugs were undetectable (100 % removed) in the effluent. They were: **Analgesic**: Paracetamol, Salicylic acid, Oxycodone; **Anti**-**inflammatory**: Naproxen + ve, Atorvastatin, Indomethacin, Naproxen; **Alkaloid**: Caffeine; **Lipid**/**cholesterol lowering**: Gemfibrozol.

**Conclusions:**

The study results revealed that the micropollutants removal through this biological treatment process was similar to previous research reported from other countries including Europe the Americas and Asia, except for acesulfame, a highly persistent artificial sweetener. Surprisingly, acesulfame was diminished to a much greater extent (>90 %) than previously reported research for this type of WWTPs (45–65 %) that only include physical removal of objects and solids and a biodegradation step.

## Background

Health and environmental concerns about the effects of micropollutants in wastewater have become increasingly important in wastewater management. The term micro-constituents includes pharmaceuticals and personal care products (PPCPs), and other compounds that may be found in wastewater in small amounts including compounds used in food processing and agriculturally used pesticides. Public concern increases particularly in situations where wastewater effluent is released into the environment (e.g., streams and rivers) that are then used as a raw potable water source for communities located downstream [1]. This especially concerns countries in Europe, where recycled water is used not only for agricultural purposes but also for preparation of drinking water. Water usage has become highly critical in other countries as well. For example, several states in Australia are committed to recycle more water in the future due to Australia’s general arid climate, frequent droughts and other pressures. Local governments are searching for strategies to minimise micropollutant release into surface waters and/or increase of removal from wastewater, in order to ensure the health of humans and their ecosystem [2].

As of 2006, there are about 50,000 chemicals used for industrial, agricultural and veterinary purposes in Australia [3]. Since the early 1990s, chemical assessments have taken place which resulted in the creation of different strategies and regulations for the utilisation and manipulation of pesticides, medicines, and so forth [4]. The National Industrial Chemicals Notification and Assessment Scheme (NICNAS) and the Therapeutic Goods Administration are examples of regulators created to control the use and disposal of industrial chemicals and pharmaceuticals, respectively. Significant questions remain about the types and levels of monitoring of treatment processes required in order to adequately protect human health and the environment.

The three largest sources of PPCPs include industry, hospitals, and private homes. PPCPs enter into the sanitary sewer primarily through excretion of un-metabolised pharmaceuticals [5]. Table 1 shows urinary excretion percentage as parent compound of some of the most common pharmaceuticals found in sewerage systems. In addition, residual products are frequently discarded via the sewerage system. For example, the results from a survey of the American public found that only 1.4 % of the surveyed people returned unused medication to the pharmacy, whereas 54 % threw them away and 35.4 % disposed them in the sink or the toilet [6]. Another source of these compounds are uncontrolled landfill sites where they and their chemical or biological degradation products reach nearby rivers or groundwater as surface run off or leachate [7]. Surface run-off may also contain chemicals from agricultural activity such as pesticides and animal medicines [8].

**Table 1:** Percentage of drug found in urinary excretion as parent compound for common medicines

Drug	Class	Compound excreted (%)	References
Ibuprofen	Non-steroidal anti-inflammatory (NSAID)	10	[53]
Paracetamol	Painkiller	4	[54]
Erythromycin	Antibacterial	25	[54]
Sulfamethoxazole	Antibacterial	15	[55]
Atenolol	β - blocker	90	[53]
Metoprolol	β - blocker	10	[54]
Carbamazepine	Antiepileptic	3	[54]

Recently, artificial sweeteners (ASs) have been identified as emerging environmental contaminants [9–13]. ASs are widely used in foods, particularly beverages, as sugar substitutes and are excreted mainly unmetabolised. They are excreted via the kidney and reach surface waters of the environment mainly in this unchanged form [11–13]. Acesulfame (ACE) is one of these sugar substitutes found in the aquatic environment as a result of effluents containing ACE from wastewater treatment plants being discharged into water courses. Acesulfame concentrations in the wastewater treatment plant effluents were reported from as low as 20 μg/L [14] up to very high values as 2.5 mg/L [11–13, 15]. It is so persistent that on the one hand, it has raised concern as an environmental pollutant but on the other hand, it is appreciated as a marker for contamination of e.g., groundwater with domestic wastewater [12, 16]. However, as Lange et al. [12] identified the actual knowledge about environmental persistence and potential hazard of ACE is not well understood.

The impact of most micropollutants on human health and environment is not well understood. However, effects of some PPCPs on human health and environment are known. For example triclosan, an antibacterial and antifungal used in personal care products such as soap and tooth paste, is called an endocrine disrupting compound (EDC) because it interferes with natural hormonal functions, potentially altering metabolism, development, reproduction and growth [17] including decline in reproductive function in men [18]. Effects of EDCs on the environment comprise birth abnormalities and feminisation of organisms including fish, frogs, birds and mammals [4]. Effects on aquatic organisms have also been documented for certain herbicides, such as 2,4-Dichlorophenoxyacetic acid (2,4 D), which alters the shell formation of the bivalve *Anodonta cygnea* [16]. This compound has also been investigated intensely for chronic toxicity in humans but results remained inconclusive [19].

Some water micro-constituents, such as the antibiotic ciprofloxacin and the artificial sweetener acesulfame, have been shown to be degraded during certain treatment steps, which can lead to conversion into more toxic compounds [9, 20]. Antibacterials are of concern not only because of their toxicity but also as harbinger of bacterial resistance. Bacteria in wastewater comprise very high levels and varieties of resistance genes, which may be disseminated to human and animal pathogens [21–23]. Multi-resistance in bacteria is a major global health issue that has further restricted treatment options for already limited options of infectious diseases [24].

There is only limited knowledge around the accumulative effects of individual compounds and combination effects of mixtures of micropollutants in wastewater. Some advances in this field have been made with the consideration of toxic equivalent concentrations and the use of mode of action based test batteries where concentrated water samples are tested and the risk is assessed by comparing the results of e.g., environmental water samples to specific reference compounds for each test [25–27]. Advanced methods of water treatment have been designed for reclamation of wastewater for drinking purposes including reverse osmosis, ozonation, UV-irradiation, nano- and ultrafiltration, activated carbon filter and biofiltration. When applied after traditional wastewater treatment these techniques reduce a wide variety of biological effects including estrogenic, genotoxic, neurotoxic and phytotoxic effects. These reduction of these effects varied depending on the treatment combined with the compound composition of the water [28–30].

In summary, it becomes clear that additional steps for wastewater treatment are essential to decrease the discharge of micropollutants [31] into Australian rivers and estuaries as current traditional wastewater treatment is insufficient to avoid their release into the environment. In South-East Queensland, several studies have investigated the removal of micropollutants in modern state of the art water reclamation plants [27–30]. These studies have focussed on the removal of micropollutants with sophisticated methods. Furthermore, in the South-East Queensland region, Shareef et.al [32] investigated the removal of EDCs and PPCPs at Oxley Creek and Luggage Point which received wastewater from domestic and domestic/industrial sources respectively. Ying et al. [33] studied the estrogens and xenoestrogens removal in the final effluents of five wastewater treatment plants from South-East Queensland. Also, Tan et al. [34] did a comprehensive study of the removal of 15 EDCs and estrogen equivalent (EEqs) of five wastewater treatment plants from South-East Queensland. Two other studies have investigated the fate of antibacterials and these were used for comparison where appropriate [35, 36].

This paper assesses the efficiency of conventional wastewater management practices on the removal of PCPPs and other micropollutants in a traditional 3-step WWTP in South-East Queensland to inform future individual wastewater management plans for this WWTP. The concentrations of 95 different micropollutants in wastewater influent and effluent were determined. Removal rates for the biodegradation step were calculated for compounds that were found in the influent.

## Methods

### Description of the wastewater treatment plant

The municipal wastewater treatment plant (WWTP) receives wastewater mainly from households, hospitals and various manufacturing and service industries including meat manufacturing, automobile repairing and maintenance, fuel retailing, laundry and dry cleaning and spirit manufacturing.

**Fig. 1:**
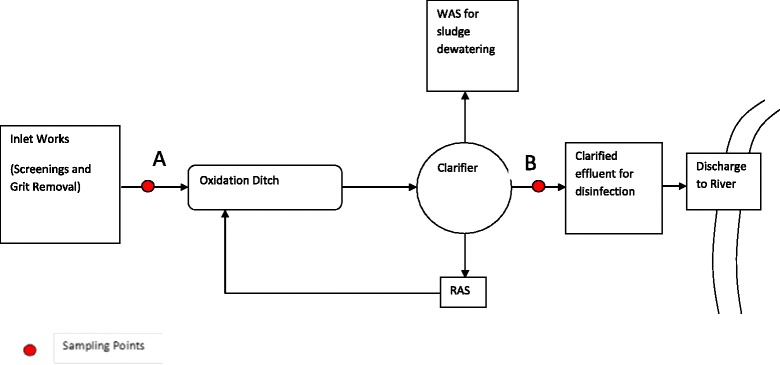
Flow diagram of the studied wastewater treatment plant

The WWTP is equipped with a conventional 3-step treatment process including physical removal of objects and solids, biological oxidation and a chlorine based disinfection process (Fig. 1). Preliminary treatment involves three band screens and two grit chambers, which removes grit and screenings. Sodium hypochlorite (NaOCl) and sodium hydroxide (NaOH) are used in the preliminary treatment for odour control and pH adjustments. The system has a fully automated wet weather by pass system to send excess flow into environment (a receiving stream). The biological process integrates four oxidation ditches (OD) with an aerobic and anaerobic zone followed by clarifiers. The sludge dewatering facility consists of a gravity drainage deck (GDD) and a belt filter (BFP) process. The disinfection process uses chlorination. Current average flow rate of influent is 45 ML/day.

Sampling points are shown in Fig. 1 as A and B. Sampling Point A: The wastewater influent, the raw wastewater, was collected after screenings and grit removal. Sampling Point B: The effluent was collected after the biological oxidation ditch and clarifier, but before disinfection of the final effluent. WAS: Waste Activated Sludge, RAS: Return Activated Sludge.

### Sampling and analysis

During September 2012, grab samples were collected in alignment with the Water Monitoring Data Collection Standards of Queensland Government using the methodology described in AS/NZS 5667.1 Water Quality – Sampling – Guidance on the design of sampling programs, sampling techniques and the preservation and handling of samples [37–39]. Two sets of samples were collected at two different locations (influent A and effluent B) of the WWTP on 2 different days. The sampling points were located at two different stages of the treatment plant process: Point A was located after primary treatment and before entering the biological treatment unit (oxidation ditch), while Point B after the biological treatment and clarification process (Fig. 1). In total eight grab samples were collected in 500 ml plastic bottles following the hydraulic retention time (average HRT = 24 h; i.e., 20 h in oxidation ditch and 4 h in clarifier) of the WWTP and together with blank samples were stored at 4 °C until analysis.

The samples in this study were analysed for a total of 95 compounds, including PPCPs, agricultural, food processing and other micropollutants, which are commonly found in wastewater, using an in-house validated analytical method from Queensland Health Forensic and Scientific Services. This method has been optimised according to the USEPA Method 1694 [40]. A 1 mL filtered-sample was spiked with mass-labelled chemical standards (compensating for any instrumental variations during analysis) and analysed using liquid chromatography (Shimadzu Prominence, Shimadzu Corp., Kyoto, Japan) coupled with Tandem mass spectrometry (LC-MS/MS; Applied Biosystem/Sciex API 4000Q system). Separation of the compounds was performed on a C18 analytical column (Luna C18, 150X2.1 mm, 3 μm, Phenomenex) at 45 °C with a gradient mobile phase (A: 1 % acetonitrile, 99 % Milli-Q water and 0.1 % formic acid; B: 95 % acetonitrile, 5 % Milli-Q water and 0.1 % formic acid) programmed as: 8 % B at start; ramped up to 35 % B at 3.5 min; increased to 100 % B at 11 min; held 100 % B for 4 mins; equilibration of the column for 3 mins. The mass spectrometry was operated in a multiple reaction monitoring mode to identify and quantify the micropollutants in the samples.

## Results

Wastewater samples were taken at two sampling points before and after the biodegradation step, which has an aerobic and an anaerobic zone and analysed 95 compounds including PPCPs, compounds used in food processing and agriculture by LC-MS/MS. From the two sets of samples collected on two different days (with 5-days apart), during the month of September 2012, the arithmetic average pollutant levels of the two samples were calculated. Out of the 95 compounds tested, we found 38 compounds in the influent, mainly drugs or drug metabolites as shown in Table 2. There were three pesticides and two food components, the artificial sweetener acesulfame and caffeine. Nine of the identified compounds were undetectable in the effluent, but 29 were still present to various degrees in the effluent samples after the biodegradation step in the oxidation ditch.

**Table 2:**
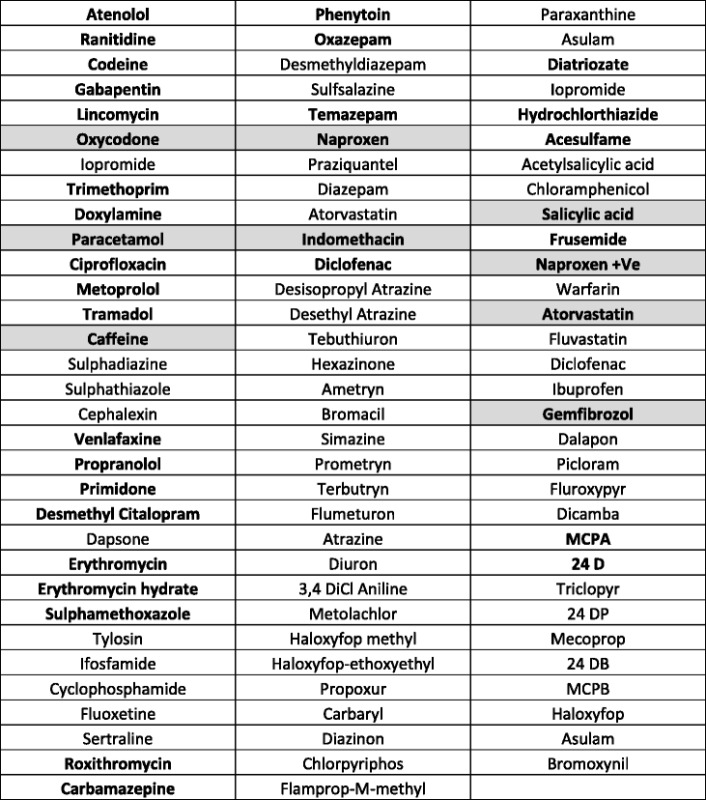
List of 95 compounds tested in the influent (The 38 compounds detected in the influent are in bold with 9 compounds absent in effluent shaded)

**Fig. 2:**
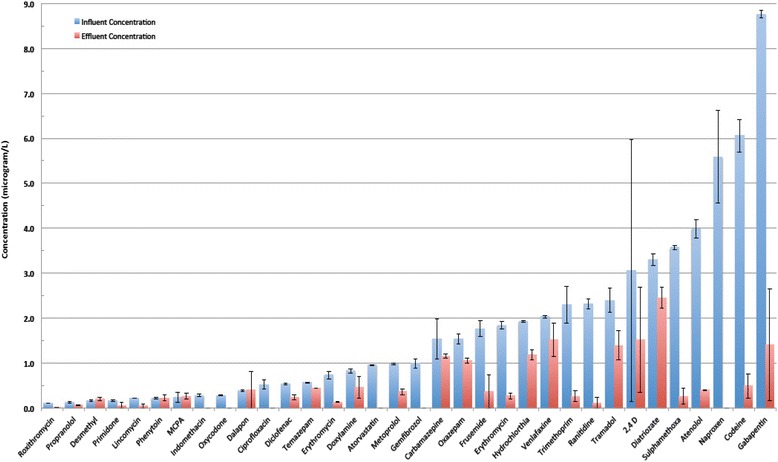
Removal of micropollutants with low to medium levels of pollutant in the influent (Mean with error bars depicting minimum and maximum observed values)

**Fig. 3:**
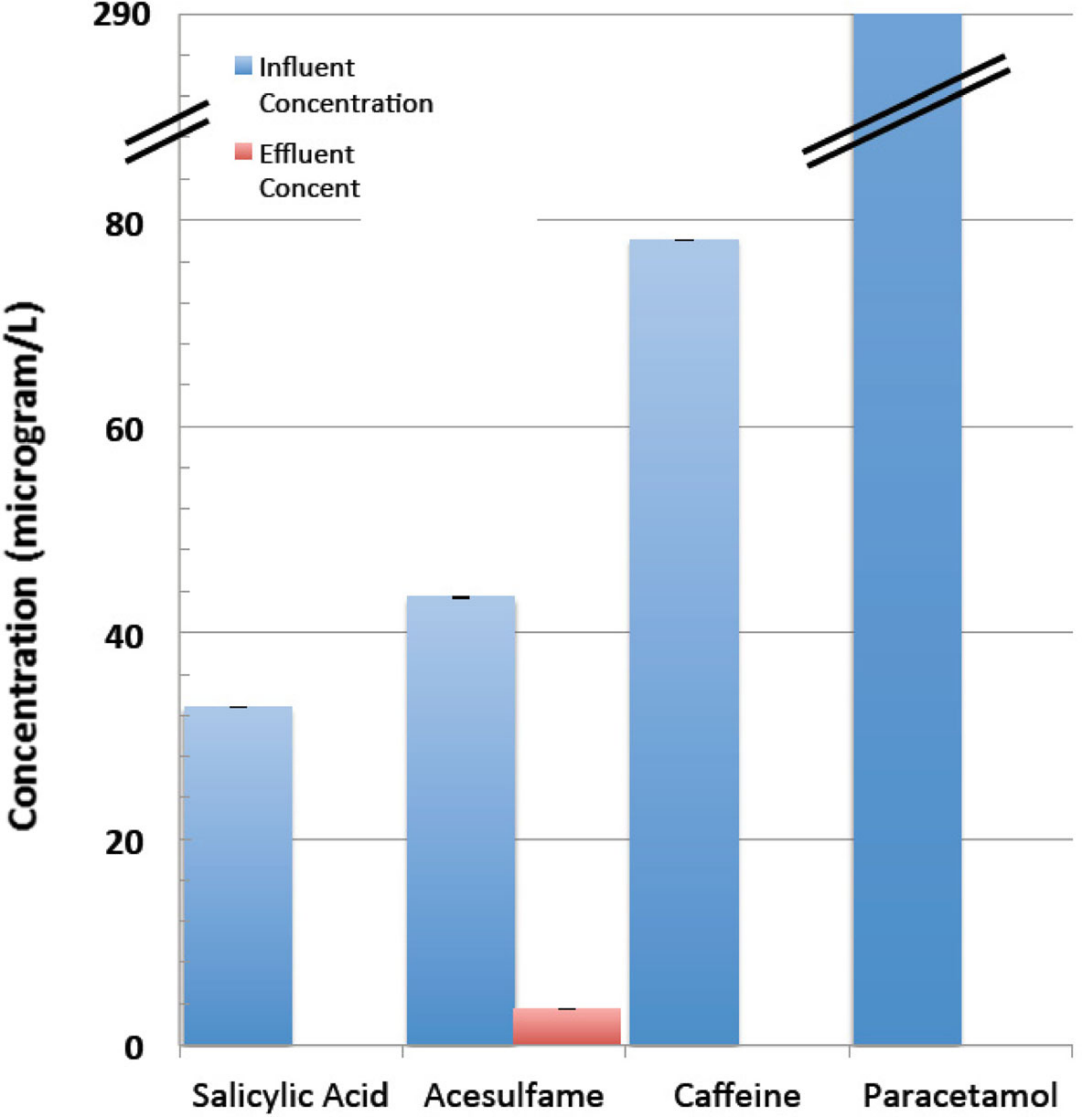
Removal of micropollutants with high levels of pollutant in the influent (Mean with error bars depicting minimum and maximum observed values)

The majority were removed to at least 80 % (Fig. 2). The most persistent compounds included MCPA (2-methyl-4-chlorophenoxyacetic acid), 2,4-D (2,4-Dichlorophenoxyacetic acid), desmethyl citalopram, phenytoin and carbamazepine (Fig. 2). Surprisingly, acesulfame was removed in the conventional treatment process by 92 % (Fig. 3). Three compounds paracetamol, salicylic acid and caffeine found at concentrations of 289.40 μg/L, 32.74 μg/L, and 78.09 μg/L respectively in the influent were undetectable in the effluent (Fig. 3).

Tables 3 and 4 present the characteristics of raw wastewater (influent) received by the treatment plant and the quality of treated effluent before disinfection and discharge respectively. The data are typical for water quality of this type of traditional treatment plants. The treatment plant total nitrogen (TN) discharged in the adjacent licensed discharge point is 53 t per year, keeping acceptable levels according to the maximum permitted by regulator (Environment and Heritage Protection) for the river (98 t per year).

**Table 3:** Inlet wastewater quality

Date	pH	NH_3_ mg/L	NOx-N mg/L	ortho-PO_4_-P mg/L	TP mg/L	TN mg/L	TSS mg/L	TKN mg/L	COD mg/L	BOD mg/L	Alkalinity mg/L (CaCO_3_)
3/09/2012	7.5	66	0.2	7.7	8.7	71	330	71	760	460	340
5/09/2012	7.1	74	0.1	7.7	8.2	76	370	76	800	440	350
10/09/2012	7.9	49	0.4	7.0	7.0	73	560	73	750	620	330
12/09/2012	7.5	54	0.2	8.0	11.0	87	580	87	1180	700	370
17/09/2012	7.6	46	0.1	6.8	10.0	70	390	70	830	480	340
19/09/2012	7.6	58	0.1	7.4	7.9	83	950	83	950	618	370
24/09/2012	7.7	50	0.1	6.9	10.0	69	420	69	810	540	370
26/09/2012	7.5	63	0.1	7.7	10.0	80	510	80	960	540	370

*TN* total nitrogen, *TP* total phosphorus, *TSS* total suspended solids, *TKN* total Kjedahl nitrogen, *COD* chemical oxygen demand, *BOD* biochemical oxygen demand

**Table 4:** Quality of treated effluent after clarification

Date	NH_3_ mg/L	NOx mg/L	ortho PO_4_-P mg/L	pH	TSS mg/L	TP mg/L
3/09/2012	2.1	0.7	3.2	7.6	4	3.4
5/09/2012	1.7	0.8	3.7	7.6	5	3.9
10/09/2012	0.8	0.7	2.9	7.8	6	3.1
12/09/2012	0.7	0.7	4.3	7.6	6	4.6
17/09/2012	0.9	0.7	4.1	7.7	7	4.2
19/09/2012	0.1	1.8	5.6	7.5	3	5.8
24/09/2012	0.4	0.4	2.6	7.7	7	2.8
26/09/2012	0.3	0.7	3.4	7.7	7	3.6

*TN* total nitrogen, *TP* total phosphorus, *TSS* total suspended solids

## Discussion

The concentrations of observed micropollutants in the wastewater of the investigated WWTP in South-East Queensland were generally within the lower range of previously reported values from around the world [36, 41–45]. This is in agreement with the comparatively low population density in the catchment of the observed WWTP. Where applicable, all values were at least one order of magnitude below the Australian Drinking Water Guidelines 6 [46] and the Australian Guidelines for Water Recycling [47]. In addition, the differences between concentrations of micropollutant parent compounds in influent and effluent water observed in our study fit well within the ranges of current reports from other studies [12, 43].

In the present study, caffeine is at about 80 μg/L in the influent samples. This is about 10 times higher than the study by Shareef et al., (8 μg/L = 8000 ng/L) [32]. The difference is reasonable due to the difference of business and human activities, lifestyle, and probably season of the year. Our studied WWTP serves the catchment with about 180,000 people (2011 census). For Oxley and Luggage point, the WWTPs serves about 85,000 and 300,000 people respectively.

With an influent concentration of about 43 μg/L, the surprisingly higher reduction (about 92 %) of acesulfame at the Queensland WWTP is the first report of reduction of this artificial sweetener in a traditional 3-step WWTP at this high level. The QA/QC details are presented below:

- Internal standard recovery: 100 % ± 3.16 % (mean ± S.D.) for caffeine and 103 % ± 6.84 % for acesulfame.
- Variation of duplicate analysis: <10 % variation (CV%) for both caffeine and acesulfame.
- Inter-day variation: <20 % (CV%) for caffeine and <5 % for acesulfame.

Milli-Q blank samples were included in the batch of analysis. No contamination of these chemicals was found in these blank samples.

Removal rates vary from study to study, which is anticipated as they depend on various conditions including the physico-chemical properties of the compounds and the treatment process itself [12, 43]. As noted earlier, acesulfame concentrations in the wastewater treatment plant effluents were reported from as low as 20 μg/L (Scheurer et al., 2009) [14] up to higher values as 2.5 mg/L (Loos et al., 2013) [15]. In the German study [14] acesulfame concentration in the influent to the wastewater treatment plant ranged from 34 to 50 μg/L and up to 41 % removal was observed.

Some compounds are biodegraded by bacteria and in these cases the removal rates can depend on their initial concentration as the removal depends partially on enzyme kinetics [42]. These effects are, however, unlikely to explain sufficiently the extremely high removal rate of ACE found in this study. In previous studies, ACE was removed consistently at 45–65 % by conventional WWT and only additional treatment with ozone or UV light increased the removal rate up to 90 % [10, 16, 40, 48]. But ozonation and/or UV light treatment of wastewater as currently practiced in water treatment plants, which prepare drinking water in Switzerland and Germany, reduced the amount of ACE by only 30 % [10, 41, 48]. The detection of a significant amount of unexpected compounds in the wastewater system, in particular the anomolous level of acesulfame removal suggests the need for a detailed assessment. Further investigation of these should use improved analytical protocols.

Our findings also raise questions with regard to the impact of the remaining ACE and its water-soluble degradation products on the environment as well as downstream users of the surface waters. However, degradation of ACE depends on the decomposition process and conditions and many transformation products that have been identified from different processes [9, 10, 48]. It is important to investigate whether the degradation products of ACE under conventional WWT in SE-Qld conditions are identical to the intermediates and products found in these studies and to assess their toxicological impact on humans and the environment.

While ACE has been substantially tested for its lack of adverse effects to humans before its registration as food additive, only limited data are available about its ecotoxic potential. Ecological toxicity tests using duckweed, *Lemna minor*, green algae, *Scenedesmus vacuolatus*, and water fleas, *Daphina magna,* revealed very high Lowest Observed Effect Concentrations (LOEC) for ACE [49].

In recent years, detection of micropollutants in wastewater and surface waters has inspired the search for practicalities beyond monitoring of their discharge into ecosystems. Monitoring the use of specific drugs, including illicit drugs, through wastewater analysis in regions and countries has become common, for example to estimate illicit drug use per capita or to monitor increases over holiday periods [50, 51]. Individual micro-constituents, such as the drugs valsartan acid, carbamapezine and the artificial sweetener acesulfame, were identified to have physico-chemical properties, which makes them resistant to degradation in wastewater treatment plants. This persistence has led to the concept of using them as tracers and markers in water bodies e.g., for identification of groundwater contamination with urban wastewater [52]. In fact, removal rate of carbamazepine in our study was also poor (~20 %). In light of our results with acesulfame it appears relevant to consider environmental conditions on local removal efficiencies of such tracers for appropriate interpretation of quantitative results in particular.

## Conclusions

The presence of 95 common micropollutants of domestic, industrial and agricultural, including pharmaceuticals, personal care products (PCPPs) and food components was determined in the wastewater influent and effluent of a particular WWTP in SE-Qld. Thirty eight compounds were found in the influent. Although this is a relatively simple conventional wastewater treatment, the levels of most of these chemicals were reduced similarly to the extent in more sophisticated WWTP, particularly regarding anti-inflammatory drugs, analgesics and antibacterials. Surprisingly, more than 90 % of the artificial sweetener acesulfame was removed in this WWTP. This anomolous level of acesulfame removal suggests the need for a detailed assessment.

Nine of the compounds in the categories of analgesic, anti-inflammatory, alkaloid and lipid/cholesterol lowering drugs were undetectable (100 % removed) in the effluent. They were: **Analgesic**: Paracetamol, Salicylic acid, Oxycodone; **Anti**-**inflammatory**: Naproxen + ve, Atorvastatin, Indomethacin, Naproxen; **Alkaloid**: Caffeine; **Lipid**/**cholesterol lowering**: Gemfibrozol.

## Abbreviations

ACE: Acesulfame
AS: Artificial sweeteners
EDC: Endocrine disrupting compounds
HRT: Hydraulic retention time
LC-MS/MS: Liquid chromatography coupled with Tandem mass spectrometry
PPCPs: Pharmaceuticals and personal care products
WWTPs: Wastewater treatment plants
